# Data-Augmented Deep Learning Models for Abnormal Road Manhole Cover Detection

**DOI:** 10.3390/s23052676

**Published:** 2023-03-01

**Authors:** Dongping Zhang, Xuecheng Yu, Li Yang, Daying Quan, Hongmei Mi, Ke Yan

**Affiliations:** 1 Key Laboratory of Electromagnetic Wave Information Technology and Metrology of Zhejiang Province, China Jiliang University, Hangzhou 310018, China; 2 Department of Building, School of Design and Environment, National University of Singapore, Singapore 119077, Singapore

**Keywords:** data augmentation, object detection, road manhole cover, deep learning, convolutional neural network

## Abstract

Anomalous road manhole covers pose a potential risk to road safety in cities. In the development of smart cities, computer vision techniques use deep learning to automatically detect anomalous manhole covers to avoid these risks. One important problem is that a large amount of data are required to train a road anomaly manhole cover detection model. The number of anomalous manhole covers is usually small, which makes it a challenge to create training datasets quickly. To expand the dataset and improve the generalization of the model, researchers usually copy and paste samples from the original data to other data in order to achieve data augmentation. In this paper, we propose a new data augmentation method, which uses data that do not exist in the original dataset as samples to automatically select the pasting position of manhole cover samples and predict the transformation parameters via visual prior experience and perspective transformations, making it more accurately capture the actual shape of manhole covers on a road. Without using other data enhancement processes, our method raises the mean average precision (mAP) by at least 6.8 compared with the baseline model.

## 1. Introduction

As an integral part of the road, the working condition of manhole covers is of great importance to the safety of drivers and pedestrians. As cities continue to expand in size, manhole covers are becoming more widespread and numerous, making manual supervision more difficult.

In recent years, deep learning methods [1,2,3] have been increasingly applied to object detection [4,5,6,7]. Thus, attention has been paid to the automatic detection of abnormal manhole covers [8,9]. Vehicles equipped with video cameras have great potential for anomalous manhole cover detection. Different from traditional methods [10,11], object detection models based on state-of-the-art conversational networks require a lot of training data. However, using vehicle cameras to collect data, we found very few anomalous manhole covers on the carriageway, far less than the number needed to train the model.

To tackle this problem, we focus on using data augmentation methods [12] to improve the efficiency of abnormal manhole cover detection data. Copy–paste augmentations can create copies from dataset samples and then paste them into other samples, which can alleviate the shortcomings of the original dataset. When using this data augmentation method, we can adjust the hyperparameter, such as the number of pasted objects from the source image and the extent of scale jittering, to find the most effective way to train our deep learning model. Prior work [13] uses randomly pasted object samples or models the surrounding visual context to decide the location and size of pasted object samples. In contrast, we find a new strategy of using perspective transformation and segmentation to decide upon the shape and size of manhole covers before being pasted to the target image, providing significant boosts on object detection models for the manhole cover detection task.

In this paper, a new data augmentation method based on the copy and paste method for road abnormal manhole cover detection is proposed. The proposed method is evaluated on an abnormal manhole cover dataset made by ourselves:

- A sample expansion method for the abnormal manhole cover dataset is proposed. This method allows obtaining a variety of anomalous coverage samples from images using geodetic information and perspective transformations to provide samples for subsequent data augmentation.
- Using extracted abnormal manhole cover samples, we proposed a visually guided copy–paste data augmentation method for abnormal manhole covers, namely VGCopy-paste. This method combines prior visual and spatial information to more intuitive paste anomalous manhole cover samples onto the image, alleviating the problems of sample imbalance and an insufficient number of samples during training.
- Better performance under different training configurations and epochs compared with the current state-of-the-art object detection models: The experimental results show that networks using the data enhancement method in this paper have higher accuracy and faster convergence than networks that do not use this method with the same configuration.

## 2. Materials and Methods

### 2.1. Data Augmentation for Deep Learning

Object detection is crucial in many downstream tasks. Detecting various objects on the road, such as pedestrians [14], vehicles [15], traffic signs [16], road markings [17], etc., in high-resolution images from the vehicle’s camera is necessary to deploy self-driving cars safely. The total loss of the model in the verification set should be gradually reduced as training proceeds to train a deep learning model with sufficient generalization ability. Many efforts to improve model performance are centered on changing the architectures of the backbone, which may lead to an increasing number of parameters with respect to the model and make it more challenging to train. In addition to increasing the complexity of the model, image data augmentation uses the semantic invariance of an image to introduce a priori knowledge via random horizontal flipping, color jittering, random crop, and other methods of the original image to improve its performance.

The above image transformation will not affect other images in the dataset, and no additional objects will be added to the transformed image. Mixing images is another kind of data augmentation. Its main idea is that the artificial generalization of training data is realized by mixing the two images. Inoue proposed Mixup [18], randomly picking two images, A and B, that are randomly flipped horizontally from the training set and then calculating the mean value of the two images. Then, the two images are mixed up in the color channel dimension, and A-labels are only used for training. Mixup will make the training samples unnatural and obtain an unclear class to training samples in the object detection task, which may lead to model confusion. Sangdoo Yun et al. proposed the CutMix [19] method. Similarly to the Mixup method, they mixed the two images from the training set. The difference is that instead of mixing at the pixel level, they replaced the original image with a sample block of another image. In the same way, the Mosaic data augmentation [20] method proposed by Alexey Bochkovskiy picks multiple different images and puts them together into one composite image after a random crop. It increases the diversity of images, enriches the image’s background, and improves the batch size in disguise during training. It is also not very friendly to datasets with many small objects. Combining augmentations that have no object awareness can result in massively inflated dataset sizes. In the case of limited training data, it may lead to overfitting.

Copy–paste augmentation and CutMix have something in common. They both paste the target from other images onto the original image. The difference is that the former only copies the precise pixels of the object and not the entire rectangular area containing the object and part of the background. Both Nikita Dvornik et al. [21] and Georgios Georgakis et al. [22] extracted the semantics in the image scene by training a deep learning model to determine the pasting position of the object. InstaBoost [23] also trained a deep learning model to extract contextual information from images. However, it does not copy the object from other images but only copies and pastes the existing object in the current image. Golnaz Ghiasi et al. adopted the method of randomly selecting positions to paste objects. They did not model the surrounding context and directly synthesized the targets in different backgrounds into one image regardless of whether the relative size and position of the objects are intuitively appropriate. Unlike [21], we model the context and consider the actual size and angle of the manhole covers pasted on the image.

### 2.2. Deep Learning Manhole Cover Detection

The traditional inspection method for road manhole covers is usually on-site manual inspection. This method has many potential safety hazards during rush hours. With the development of the lidar system, the method is becoming more integrated and multifunctional. Zhanying Wei et al. [24] used multiple cameras arranged symmetrically and combined with high-density lidar to obtain high-density point clouds and ultra-high-resolution images on the ground. They detected the manhole covers by combining the histogram of oriented gradients (HOGs) descriptor with symmetry features and support vector machine (SVM). However, mobile lidar and multiple cameras are very expensive, and it takes a long time to process high-precision images, resulting in a long period of manhole cover detection. Haotian Ren et al. [25] improved yolov4 and proposed a manhole cover detection method by integrating image depth information. Due to the lack of training data, the model is trained using images that are crawled from the Internet, and the quality of the images is uneven, resulting in the inability of this kind of data in training a sufficiently robust model. Baoding Zhou et al. [26] used a mobile phone fixed on the vehicle to shoot manhole covers, used the accelerometer and gyroscope of the mobile phone to record the vibration experienced by the vehicle when passing the manhole covers, and then calculated the instantaneous acceleration. They trained a model that can judge the settlement amplitude of the manhole covers by combining the two.

## 3. Data-Augmented Deep Learning Model

A normal manhole cover should be placed flush with the road’s surface while maintaining its appearance integrity. Damage to the surface of the manhole cover and a deviation in the position of the manhole cover will pose a threat to the safety of vehicles and pedestrians. To more clearly discuss the data augmentation of manhole covers, we divide the abnormal well cover into three categories, namely “Damaged”, “Dislocated”, and “Missing”. Here, “Damaged” represents cracks or extra holes in the appearance of the manhole cover, “Dislocated” represents a manhole cover that is not flush with the road’s surface, including the manhole cover bulge and depression, and “Missing” represents a manhole cover that is missing, and the road’s surface is exposed with holes. If a dislocated manhole cover is damaged, it will be classified as “Dislocated”.

**Figure 1:**
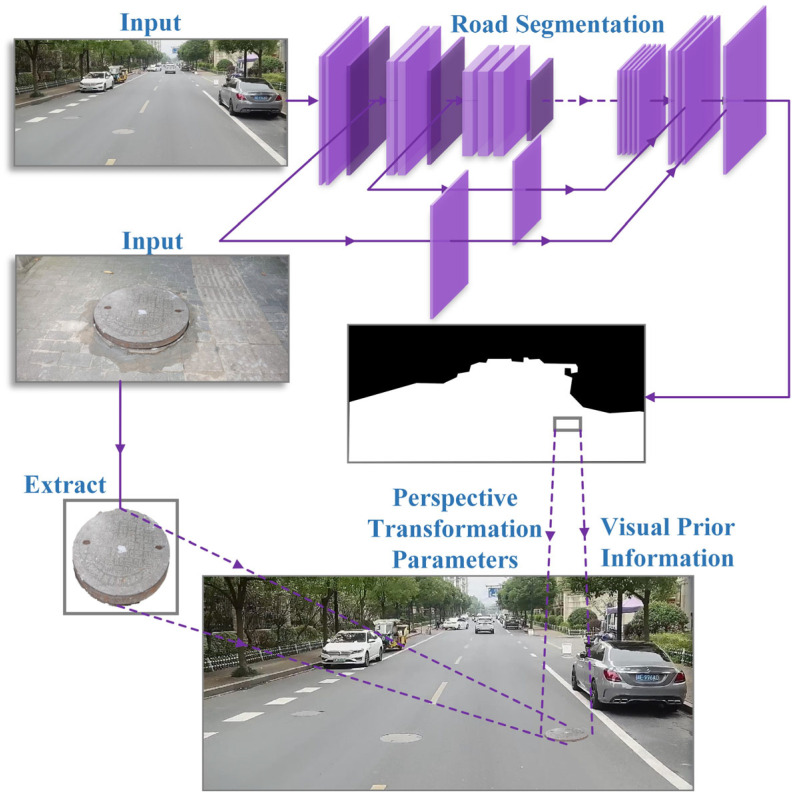
Overview of our proposed VGCopy-paste data augmentation for road manhole cover detection. We used the image taken by the vehicle’s camera and the abnormal manhole cover image taken by the mobile device as the input. Using the road semantic segmentation algorithm to obtain prior visual information, that is, the road segmentation map, we found the corresponding perspective transformation parameters for pasting and finally paste the extracted manhole cover samples onto the road.

The overall design of our method is shown in Figure 1. Two phases in the following subsections are presented: abnormal manhole cover sample expansion and visually guided copy–paste data augmentation.

### 3.1. Abnormal Manhole Cover Sample Expansion

Due to the small number of abnormal manhole covers on the road, mobile devices are used additionally to find and take images of abnormal manhole covers from multiple locations. In an effort to paste the captured manhole cover samples onto the image in the dataset, it needs to be further transformed because the viewing angle of the mobile device is different from that of the vehicle’s camera, and their visual features change with distance. However, in prior copy–paste-like works, instance segmentation masks provided in the dataset or made by us are used to make a copy of any object from the original location, and random transformations are applied. Then, the copy is pasted to other images in the training set. We suppose that the manhole cover image captured by the mobile device is directly pasted into the dataset captured by the vehicle’s camera without processing. In that case, the composite image will look unnatural, and with respect to manhole cover samples, it will be easier to introduce the background features of the original image where it is located. The model will have high accuracy in detecting the copied and pasted covers but will not work well on the actual data due to the cover’s shape, angle, and color, which is contrary to the idea presented in this paper.

The principal idea behind our algorithm is to use ellipses to fit the shape of manhole covers and use perspective transformation to transform them into regular circular manhole covers.

For restoring an irregular elliptical manhole cover to a circular one, a standard ellipse could be used to fit the boundary of the manhole cover in the image. Then, the transformation matrix is solved via point pairs using perspective transformation to restore the image to a circular manhole cover. Equations (1)–(3) show the process of projecting the object to a new view plane:

(1) $$\begin{array}{c} \left[\begin{array}{c} x^{\prime }\\ y^{\prime }\\ w^{\prime } \end{array}\right]=H\left[\begin{array}{c} u\\ v\\ w \end{array}\right] \end{array}$$

(2) $$\begin{array}{c} H=\left[\begin{array}{ccc} a_{11} & a_{12} & a_{13}\\ a_{21} & a_{22} & a_{23}\\ a_{31} & a_{32} & a_{33} \end{array}\right] \end{array}$$

where *u* and *v* are the coordinates of the object in the original image, and $H\in R^{3\times 3}$ is the transformation matrix. From the equations above, the coordinates of the object in the new view plane can be expressed as follows:

(3) $$\left\{ \begin{array}{c} x=\frac{x^{\prime }}{w^{\prime }}=\frac{a_{11}u+a_{12}v+a_{13}}{a_{31}u+a_{32}v+a_{33}}\\ y=\frac{y^{\prime }}{w^{\prime }}=\frac{a_{21}u+a_{22}v+a_{23}}{a_{31}u+a_{32}v+a_{33}} \end{array}\right.\Rightarrow \left[\begin{array}{cccccccc} p_{r1}x & p_{r1}y & 1 & 0 & 0 & 0 & -p_{r1}x\cdot p_{s1}x & -p_{r1}y\cdot p_{s1}x\\ 0 & 0 & 0 & p_{r1}x & p_{r1}y & 1 & -p_{r1}x\cdot p_{s1}y & -p_{r1}y\cdot p_{s1}y\\ p_{r2}x & p_{r2}y & 1 & 0 & 0 & 0 & -p_{r2}x\cdot p_{s2}x & -p_{r2}y\cdot p_{s2}x\\ 0 & 0 & 0 & p_{r2}x & p_{r2}y & 1 & -p_{r2}x\cdot p_{s2}y & -p_{r2}y\cdot p_{s2}y\\ p_{r3}x & p_{r3}y & 1 & 0 & 0 & 0 & -p_{r3}x\cdot p_{s3}x & -p_{s3}y\cdot p_{s3}x\\ 0 & 0 & 0 & p_{r3}x & p_{r3}y & 1 & -p_{r3}x\cdot p_{s3}y & -p_{r3}y\cdot p_{s3}y\\ p_{r4}x & p_{r4}y & 1 & 0 & 0 & 0 & -p_{r4}x\cdot p_{s4}x & -p_{r4}y\cdot p_{s4}x\\ 0 & 0 & 0 & p_{r4}x & p_{r4}y & 1 & -p_{r4}x\cdot p_{s4}y & -p_{r4}y\cdot p_{s4}y \end{array}\right]\left[\begin{array}{c} a_{11}\\ a_{12}\\ a_{13}\\ a_{21}\\ a_{22}\\ a_{23}\\ a_{31}\\ a_{32} \end{array}\right]=\left[\begin{array}{c} p_{s1}x\\ p_{s1}y\\ p_{s2}x\\ p_{s2}y\\ p_{s3}x\\ p_{s3}y\\ p_{s4}x\\ p_{s4}y \end{array}\right]$$

where *x* and *y* are the coordinates of the object in the new view plane after perspective transformation, and $\left(p_{ri}x,p_{ri}y\right)$ and $\left(p_{si}x,p_{si}y\right)$ represent the coordinates of points $p_{ri}$ and $p_{si},\;i\in \left\{1,2,3,4\right\}$, respectively. Normally, $a_{33}$ is made equal to 1 by normalization. Therefore, the perspective transformation matrix has 8 degrees of freedom. Thus, generally speaking, four points correspond to only a two-dimensional perspective transformation. Given a standard ellipse e, for obtaining the appropriate four points from the edge of the manhole cover in the picture taken by the mobile device and determining the diameter of the manhole cover after restoration, first, we fit the manhole cover in images by adjusting the long axis, short axis, and inclination angle of e; then, we use the four vertices $p_{e1},p_{e2},p_{e3},p_{e4}$ of ellipse e to construct its circumscribed rectangle r. To ensure that the resolution of the manhole cover restored to a circular shape will not cause a loss of its appearance features, we then form four pairs of points corresponding to the four vertices $\left(p_{r1},p_{r2},p_{r3},p_{r4}\right)$ of the circumscribed rectangle r and the four vertices $\left(p_{s1},p_{s2},p_{s3},p_{s4}\right)$ of the square view plane s formed by the long side of the circumscribed rectangle, and we calculate the corresponding transformation matrix, H. Finally, Equations (1) and (3) are used to project the irregular elliptical manhole cover onto the square view plane via H to form a circular manhole cover.

**Figure 2:**
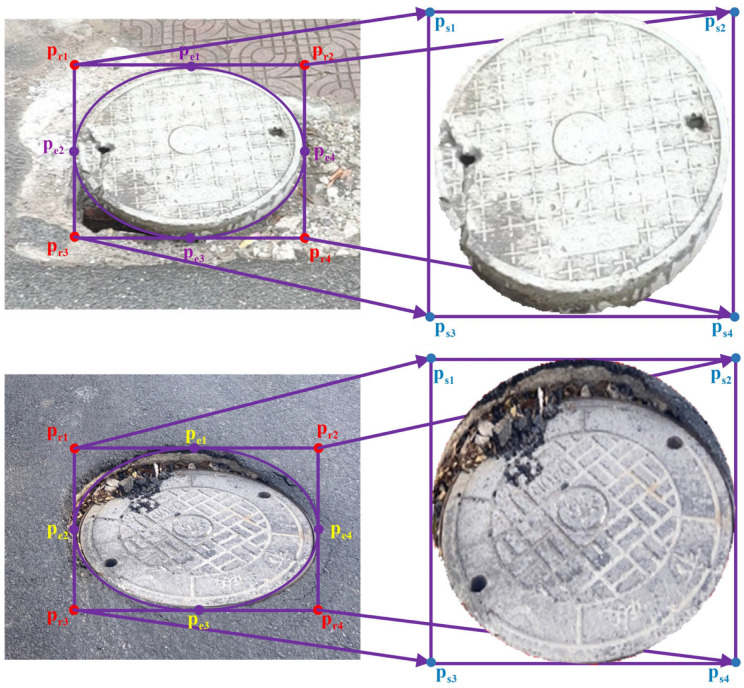
The process of recovering the shape of the manhole cover based on perspective transformation. The convex and concave abnormal well covers may have the same complete appearance as normal well covers, but they are often not aligned with the road surface. Therefore, the thickness of the convex well covers and the concave depth of the concave well covers need to be considered when extracting their samples.

The example of an image taken by the mobile device and the results of recovering the shape of the manhole cover based on perspective transformations are presented in Figure 2. The four vertices of bounding rectangle r of ellipse e and the four vertices of the square view plane form four groups of point pairs, $\left(p_{r1},p_{s1}\right),\;\left(p_{r2},p_{s2},\right),\;\left(p_{r3},p_{s3}\right),\;\mathrm{and}\;\left(p_{r4},p_{s4}\right)$, to solve transformation matrix H.

### 3.2. Visually Guided Copy–Paste Data Augmentation

In this subsection, we address the problem of pasting manhole cover samples. The major steps can be grouped into two stages: (1) the pasting method of abnormal manhole cover samples and (2) the adaptive pasting method combined with scene semantics information.

#### 3.2.1. Pasting Method of Abnormal Manhole Cover Samples

A new dataset containing only circular manhole covers was made after using matching point pairs to construct homograph matrix H and extracting the manhole cover from the original images. Due to the different devices used by collectors when taking images of the original dataset and the significant differences in the position, size, and angle of the covers in the images, the difference in size between manhole covers in the new dataset will be large. Nevertheless, in practice, the size of each type of manhole cover is fixed. The round cover samples cannot be pasted onto the images directly because the restored round manhole covers can be seen as being taken vertically from the top of the covers, and the shooting angle of the vehicle’s camera is not perpendicular to the ground.

**Figure 3:**
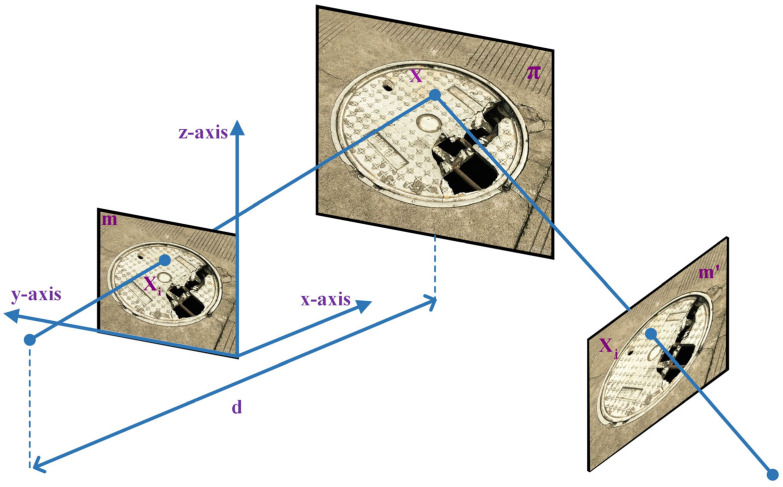
Example of a damaged manhole cover under the mobile equipment coordinate system and vehicle camera coordinate system.

A new homography matrix, $H_{2}$, will be constructed using the perspective transform to paste the circular manhole cover onto the target image. As shown in Figure 3, we suppose that m and $m^{\prime }$ are the imaging of plane π in two cameras. In this case, plane π was considered as a circular manhole cover; m as an image was taken vertically with a mobile device from the top of π; $m^{\prime }$ as an image of π was taken from the perspective of the vehicle’s camera. The unit normal vector of plane π in the mobile device coordinate system is n, and the distance from π to the center of the mobile device (coordinate origin) is d; plane π can be expressed mathematically by Equation (4):

(4) $$\begin{array}{c} \frac{1}{d}n^{T}X_{i}=1,\;\forall X_{i}\in \pi \end{array}$$

where $X_{i}$ denotes the coordinate of 3D point X in the mobile device’s coordinate system and then the coordinate of X in the vehicle camera coordinate system is $X_{j}$, which is mathematically shown in Equation (5):

(5) $$\begin{array}{c} X_{j}=RX_{i}+T \end{array}$$

where $R\in R^{3\times 3}$ denotes the rotation matrix, and $T\in R^{3\times 1}$ denotes the translation matrix. The homography matrix $H^{\prime }$ of two different camera coordinate systems in the same plane π can be obtained by combining Equations (4) and (5), and this is mathematically shown in Equation (6).

(6) $$\begin{array}{c} \left\{\begin{array}{c} X_{j}=RX_{i}+T\frac{1}{d}n^{T}X_{i}=\left(R+T\frac{1}{d}n^{T}\right)X_{i}=H^{\prime }X_{i}\\ H^{\prime }=R+T\frac{1}{d}n^{T} \end{array}\right. \end{array}$$

The $H^{\prime }$ mentioned above represents the mapping of 3D points between two coordinate systems, and it is also necessary to transform 3D points into a 2D imaging plane coordinate system. Equations (7)–(9) show the conversion of $H^{\prime }$ to $H_{2}$ using different camera internal parameter matrices:

(7) $$\begin{array}{c} \left\{\begin{array}{c} x_{i}=K_{i}X_{i}\\ x_{j}=K_{j}X_{j} \end{array}\right. \end{array}$$

(8) $$\begin{array}{c} K_{j}{}^{-1}x_{j}=H^{\prime }K_{i}{}^{-1}x_{i}\Rightarrow \;x_{j}=K_{j}H^{\prime }K_{i}{}^{-1}x_{i} \end{array}$$

(9) $$\begin{array}{c} H_{2}=K_{j}\left(R+T\frac{1}{d}n^{T}\right)K_{i}{}^{-1} \end{array}$$

where $K_{i}$ denotes the internal parameter matrices of mobile devices, $K_{j}$ denotes the internal parameter matrices of the vehicle’s camera, and $H_{2}$ denotes the homography matrix between m and $m^{\prime }$.

When processing pictures, we adjust the position of the virtual camera via rotation matrix R and translation matrix T so that it moves from the shooting direction of the mobile device to the vehicle’s camera. Equations (10) and (11) show R and T:

(10) $$\begin{array}{c} R=\left[\begin{array}{ccc} 1 & 0 & 0\\ 0 & cos\theta & -sin\theta \\ 0 & sin\theta & cos\theta \end{array}\right] \end{array}$$

(11) $$\begin{array}{c} T=\left[\begin{array}{c} 0\\ sin\theta \\ 0 \end{array}\right]+\left[\begin{array}{c} 0\\ 0\\ -d\prime *cos\theta -d \end{array}\right] \end{array}$$

where $d^{\prime }$ denotes the distance between the vehicle’s camera and the manhole cover, and i and j represent the shooting direction of the mobile device and vehicle’s camera, respectively.

#### 3.2.2. Adaptive Pasting Method Combined with Scene Semantics Information

**Figure 4:**
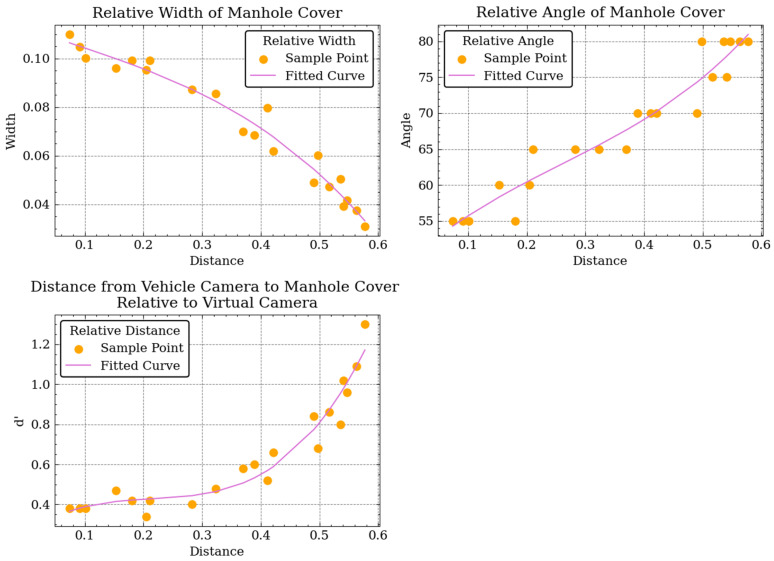
Linear fitting perspective transformation parameters, from left to right, are the width and angle of the pasted manhole cover sample and the distance from the vehicle’s camera.

While the mobile devices used by the image collector are all different, the vehicle’s camera was not calibrated before starting shooting. Since there is no depth information in the images, $K_{i}$, $K_{j}$, d, and $d^{\prime }$ cannot be directly computed. We assume that deep learning techniques are used to predict parameters based on paste positions. A large amount of data are required to train the model, which are unavailable in real-world scenarios. By observing the images taken by the vehicle’s camera, it can be found that the contour of the manhole cover located slightly away from the camera cannot be observed clearly because of the shooting angle, and only part of the manhole cover on the road can show the complete contour. Thus, the manhole cover on the lower half of the image is used for data augmentation. Parameter d is set to 1, and the perspective transformation θ, $d^{\prime }$, position, and other parameters are manually adjusted and recorded to make the pasted manhole cover similar to the original manhole cover in the image; meanwhile, we set $K_{i}=K_{j}=\left[\begin{array}{ccc} \frac{w}{2} & 0 & \frac{w}{2}\\ 0 & \frac{w}{2} & \frac{h}{2}\\ 0 & 0 & 1 \end{array}\right]$, where w represents the width of the manhole cover image, and h represents the height of the manhole cover image. As shown in Figure 4, a total of 21 groups were recorded, and the data were fitted with a least squares polynomial so that it can automatically generate the appropriate homography matrix according to the different positions in the image taken by the vehicle’s camera. In the actual operation process, we made a processing tool with a UI interface to simplify the recording and adjust the perspective transformation parameters. The speed of manually pasting manhole cover samples with tools is about 1–2 pieces per minute, which took about 30 min in total.

To solve the problem of the unnatural appearance of composite images, we followed the approach of [27] to fuse the color of the manhole cover to the color of the background image. The implementation details are described in Section 4.3.

A lightweight semantic segmentation algorithm based on deep learning for road segmentation was proposed to paste the manhole cover in the appropriate position in images automatically. The architecture of the road segmentation model is illustrated in Table 1. MobileNetV3-small is used as the backbone network, and feature fusion is performed on Unet-like decoders via skipping connections. Due to the depthwise separable convolution having fewer parameters and computation, our road segmentation model has enough depth to extract image features and maintains low parameters and high efficiency. It could cost fewer computing resources to predict where the manhole cover can be pasted. Object samples can be automatically pasted using the road segmentation model and fitted perspective transformation parameters.

The VGCopy-paste algorithm is detailed in Algorithm 1.
**Algorithm 1** VGCopy-Paste Data Augmentation for Road Manhole Cover Detection
(1)  Input the abnormal manhole cover image taken by a mobile device;(2)  Fit the cover edge with an ellipse and use (2) and (3) to calculate the H;(3)  Extract abnormal manhole cover samples;(4)  Input the image taken by the vehicle camera;(5)  Find the pasting position of the cover sample in the image taken by the vehicle camera through the road segmentation model;(6)  Calculate the $H_{2}$ by (9) to paste the sample into the image;(7)  Repeat steps 1 to steps 6 until the images in the train set are all enhanced.

## 4. Discussion

### 4.1. Experimental Data

A road manhole cover dataset was made by continuously shooting along the road with an engineering vehicle equipped with a fixed-angle camera to train the road manhole cover detection and classification model. In the experiment, Hikvision DS-TCC200 was selected as the vehicle’s camera. The acquisition frequency of the camera sensor was 50 Hz, and the shooting frequency was set to once per second.

On roads with different vegetation coverage, shadows attached to manhole covers will affect their visual characteristics. A total of 22,872 photos, including high-vegetation roads, roads around buildings, and urban highways without buildings, were collected in the dataset with the aim of making the experimental data cover all kinds of roads. In total, 82 images of abnormal manhole covers at close range in several outdoor scenes were taken by handheld phones for data augmentation.

**Figure 5:**
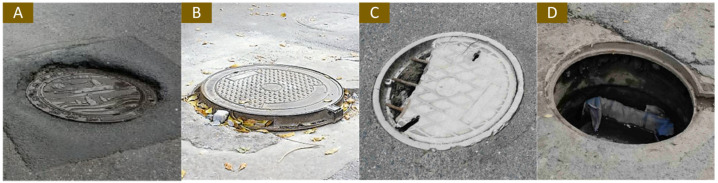
Example of three categories of abnormal manhole covers: (**A**,**B**) denote “Dislocated”, (**C**) denotes “Damaged”, and (**D**) denotes “Missing”.

**Figure 6:**
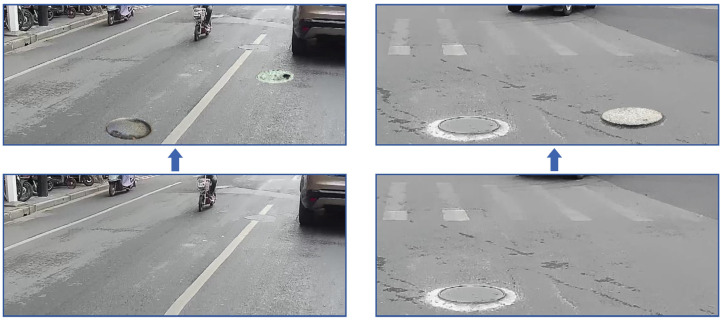
Examples of VGCopy-paste.

Figure 5 and Table 2 show the classification of manhole covers. “Dislocated” represents raised or depressed covers; “Damaged” indicates that there are cracks or holes on the surface of the covers; “Missing” indicates that the entire cover was missing, and the inspection passage is exposed; “Normal” indicates that the appearance of covers is complete and in the correct position. In total, 60% of abnormal manhole covers before executing data augmentation were used as training samples and 40% were used as testing samples. The examples of VGCopy-paste are shown in Figure 6.

UESTC All-Day Scenery [28] (UAS) is the all-day outdoor road image segmentation dataset. The entire dataset contains a total of 6380 images and four kinds of weather, including dusk, night, rainy, and sunny weather. The performance of road segmentation models was evaluated on the UAS test set.

### 4.2. Models

In our experiment, FCN [29], UNet [30], FastestDet [31], YOLOv5 [32], CenterNet [33], Retinanet [34], and YOLOv7 [35] were adopted as our baseline. As the size of the road manhole cover dataset is far smaller than the typical public dataset, FastestDet, as a lightweight network, was tested, and in all YOLOv5 series models, only YOLOv5s was tested to avoid overfitting. In addition, CenterNet with a DLA34 backbone and Retinanet with a depth of 34 were tested in our experiments. For the YOLOv7 series, the experiment only tested its basic model without expansion. FCN, UNet, and Mobile-UNet used to be evaluated on the test set of the UAS. Baselines were retrained using the corresponding open implementations. The experimental results show the impact of our data augmentation on model performance.

### 4.3. Implementation Details

The experiments are implemented in the environment built by the Pytorch deep learning framework. The only parameter modified is the training epoch. Other augmentations in the model’s configuration were not used, and the other default configurations during the experiments were provided by the authors. FastestDet, YOLOv5, and YOLOv7 were set to train 100 epochs, while CenterNet and RetinaNet trained 50 epochs. The trained models were tested using the test dataset. To compare the performance between detection models, the mean average precision (mAP) was used as the evaluation metric of model performance.

**Figure 7:**
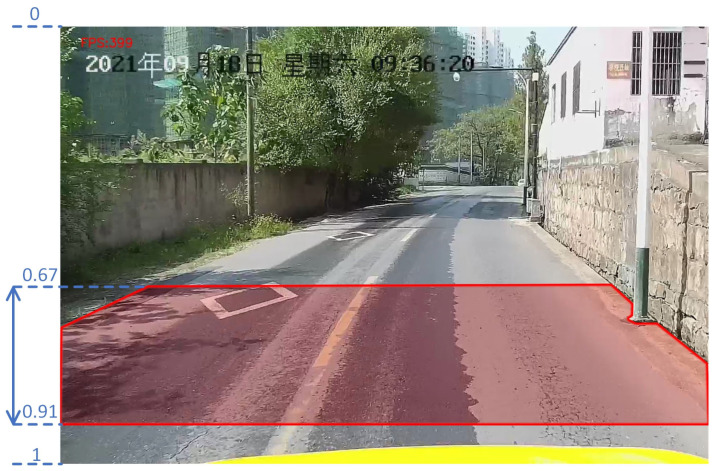
Example of pasting range of a manhole cover. The red area in the image is the pasting range.

For our VGCopy-paste, the location range where the manhole cover is pasted in images has to be set to avoid the problem of having visual features of different types of covers that are too similar due to increased distances. As shown in Figure 7, the pasting range of the manhole cover along the height direction of the background image is set from 0.67 to 0.91, and its corresponding inclination angle of covers ranges from 55 to 80 in our experiment.

Each road segmentation model was retrained on the UAS dataset for 300 epochs, and the mean IOU was used as the evaluation metric of model performance.

**Figure 8:**
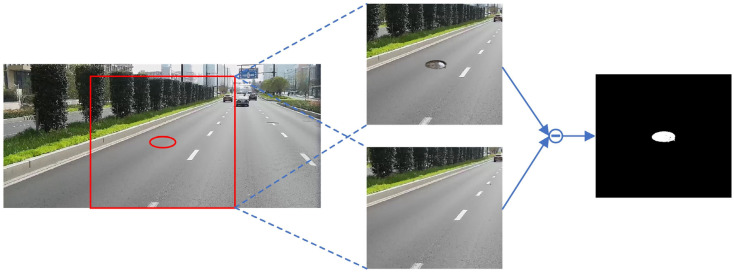
Example of image block capture and mask generation. The red ellipse represents the pasting position of the manhole cover.

When pasting the target, we used RainNet randomly to make the manhole cover blend into the background more realistically. RainNet can treat image harmonization as a style transfer problem, and we adopted a 512 × 512 resolution model, which is trained on the iHarmony4 [36] dataset. As shown in Figure 8, the resolution of the image taken by the onboard camera is 1920×1080, so the resultant image after data augmentation cannot be directly used as the input of RainNet. For distinguishing the foreground and background in the input image, we also need to provide a mask for dividing the foreground. To solve this problem, a 512 × 512 image block centered on the manhole cover was extracted as the input of RainNet, and the composite image was subtracted from the original background image to obtain the foreground mask.

**Figure 9:**
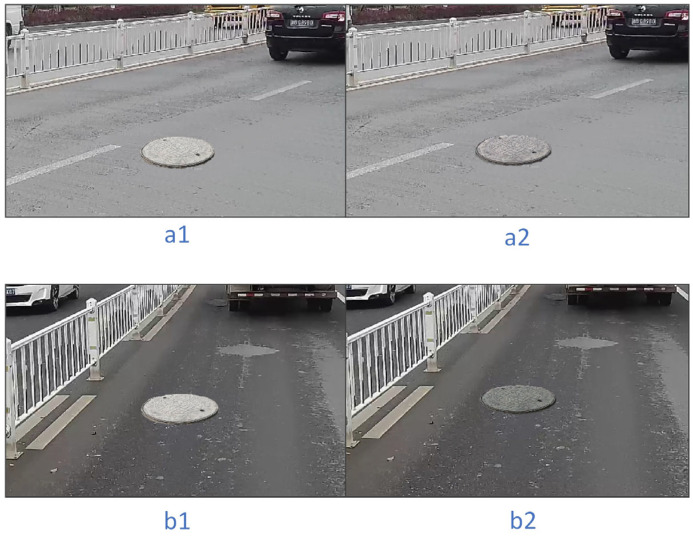
The situation of using or not using the image harmonization algorithm for the same manhole cover in different scenes, where (**a1**,**b1**) do not use the image harmonization algorithm, and (**a2**,**b2**) use the image harmonization algorithm.

Rainnet uses the foreground mask to migrate the style of the background to the foreground, and image harmonization can alleviate the incompatibility between the manhole cover pasted after data augmentation and the background. The comparison effect of Rainnet before and after use is shown in Figure 9.

### 4.4. Main Result and Analysis

The experimental results of the detection model’s performance trained using different methods are shown in Table 3, and the evaluation results of each road segmentation model are shown in Table 4. AP50 and AP75 evaluation metrics are adopted from mAP [37]. With VGCopy-past, the performance of the tested model was further improved to varying degrees during the road manhole cover detection task. The Mobile-UNet that we used performs better in the road segmentation task with fewer parameters.

Our method was compared with a simple random copy-pasting method to clarify the decisive role that prior visual experience plays in VGCopy-paste. Neither will overlap the target sample with the original sample in the image. The experiment is implemented on multiple object detection models. As shown in Table 3, when only the images taken by the mobile phone are placed into the train set without any copy–paste augmentation, the generalization ability of each model is weaker than the other two methods. Both random copy–paste and VGCopy-paste copy more samples into the dataset, and the performance of all models improved. Our method achieves better performance while increasing the same number of samples as the random copy–paste method.

In addition, we evaluated various data augmentation methods on Yolov5s, and the comparison results are shown in Table 5. Since the manhole covers only cover a small part of the image and the appearance characteristics of manhole covers in the distance are similar to the noise block introduced by Cutout, the model will be disturbed by the noise introduced. HVS augmentation can adjust the contrast and saturation of the image to make the details of the image more prominent. However, in the task of abnormal manhole cover detection, the street’s background is relatively complex, and the pavement is full of fine lines and signs. The change in background color may cause the characteristics of the manhole cover to be disturbed by the pavement’s features. Although mixup can mix multiple images to generate new images, this method does not work well for target detection tasks in complex scenes, and Random affine also does not work well. In this task, neither method can adjust the low-performance problems caused by data imbalances in the model. The method based on copying and pasting can effectively alleviate the problem of data imbalance, but random copying without controlling the pasting range will lead to model confusion, because the manhole cover is pasted to an impossible position and overlaps with the complex background.

**Figure 10:**
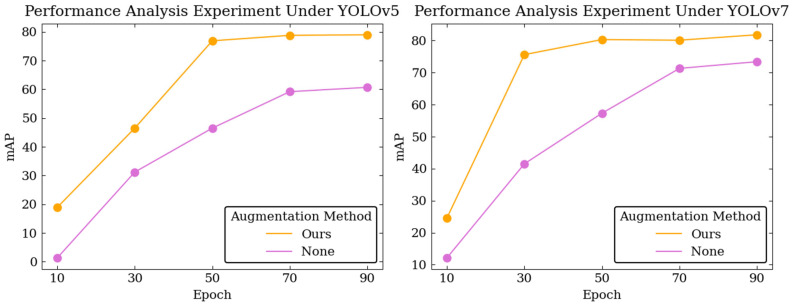
Comparison of model performance in different training epochs, where “Ours” denotes the model that used VGCopy-paste during training, and “None” denotes the model that did not use any data augmentation method during training.

Network performance was validated with and without VGCopy-paste at different training epochs to verify the role of VGCopy-paste throughout the training process. The experimental results are shown in Figure 10. VGCopy-paste can increase the training efficiency of the model. The precision of the original yolov5 and yolov7 starts to decrease at 30 epochs, and the model’s accuracy increases slowly. In contrast, after using VGCopy-paste, both models achieved better performance with less training time.

The magnitude of the enhancement of VGCopy-paste can be determined by the paste range and the number of pastes on each image. The model with different parameters was retrained, and its performance was validated on the test set; moreover, the experimental results are shown in Table 6. As the number of samples increases, the AP50 of yolov5 decreases slightly, but the performance drops significantly on the AP75. When the number of paste samples is two, yolov7 AP50 reached the best but caused four drops in AP75. The reason for this phenomenon may be that the pasted manhole cover introduces the characteristics of the original image, which changes the image distribution of the training set, and with the increase in the number of manhole covers, the difference between the image distribution trend of the training set and the distribution trend of the test set became larger; finally, the impact on the model exceeded the contribution of the manhole cover sample to the data balance. AP improved for pasted range when the range was set from 0.58 to 0.67, but it dropped rapidly as it approached 0.8. In our experiments, the number of pasted samples is set to 1, and the pasted range is set from 0.67 to 0.91. The higher the paste position, the smaller the size of the manhole cover after perspective transformations. When the manhole cover is too small, the visual characteristics of different types of manhole covers will be so similar that the model cannot effectively distinguish them by learning their appearance.

## 5. Conclusions

In this paper, a deep learning framework for abnormal manhole cover detection in urban systems is presented. A new data augmentation method was proposed to alleviate the problem of insufficient training samples for road abnormal manhole covers. After using the extraction method proposed by us to extract the manhole cover samples, we can use the simple copy and paste algorithm to greatly improve the effect of the model, as in [13]. In addition, a perspective transformation was carried out using the previous visual experience provided by road semantic segmentation and the parameters predicted by linear fitting to paste different types of abnormal manhole cover samples onto the target image so as to introduce a better performing model on the basis of random copying. According to the experimental results, the proposed data augmentation method successfully increased the number of abnormal well cover samples in the training set and subsequently enhanced the abnormal manhole cover detection performance. With the deep learning model, the mAP with AP_50_ reached over 82 and is at least higher by 6.8 compared with the baseline model using the same data augmentation model.

In the case of training, the data augmentation method based on depth learning will be more time-consuming than the traditional method. However, the combination of prior visual and data augmentation can generate the training data of abnormal manhole covers in a more intuitive way. The same sample can generate data with different appearance features in different images, which can greatly increase the data efficiency on a limited number of datasets.

In future work, we will focus on combining camera self-calibration with data enhancements based on computer vision to remove the limitation that the camera must have a fixed angle of view and achieve data enhancement for different angles in different scenes in order to further improve the accuracy of object detection tasks.

## Figures and Tables

**Table 1:** The architecture of the lightweight Mobile-UNet network.

No.	Input	Operator	Description	No. Parameters
1	512 × 512 × 3	conv2d, 3 × 3	First conv layer	464
2	256 × 256 × 16	bneck, 3 × 3	Inverted ResBlock, s = 2	744
3	128 × 128 × 16	bneck, 3 × 3	Inverted ResBlock, s = 2	3864
4	64 × 64 × 24	bneck, 3 × 3	Inverted ResBlock, s = 1	5416
5	64 × 64 × 24	bneck, 5 × 5	Inverted ResBlock, s = 2	13,736
6	32 × 32 × 40	bneck, 5 × 5	Inverted ResBlock, s = 1	57,264
7	32 × 32 × 40	bneck, 5 × 5	Inverted ResBlock, s = 1	57,264
8	32 × 32 × 40	bneck, 5 × 5	Inverted ResBlock, s = 1	21,968
9	32 × 32 × 48	bneck, 5 × 5	Inverted ResBlock, s = 1	29,800
10	32 × 32 × 48	bneck, 5 × 5	Inverted ResBlock, s = 2	91,848
11	16 × 16 × 96	bneck, 5 × 5	Inverted ResBlock, s = 1	294,096
12	16 × 16 × 96	bneck, 5 × 5	Inverted ResBlock, s = 1	294,096
13	16 × 16 × 96	shortcut + upsample	Connect to No. 12 layer	117,824
14	32 × 32 × 24	shortcut + upsample	Connect to No. 9 layer	39,376
15	64 × 64 × 16	shortcut + upsample	Connect to No. 4 layer	56,608
16	128 × 128 × 64	upsample	upsample to 512 × 512 × 2	84,800
			Total No. parameters: 1,169,168	

**Table 2:** Classification of manhole covers and the quantity of various covers collected.

Category	No. of Manhole Cover Samples Shot by the Vehicle’s Camera	No. of Manhole Cover Samples Shot by Smartphone	No. of Manhole Cover Samples after Data Augmentation
Dislocated	202	36	1549
Damaged	81	26	1035
Missing	41	20	809
Normal	4781	0	4781

**Table 3:** Road manhole cover detection on our test set, where “Without copy-paste” denotes the abnormal manhole cover images that are added to the training set without additional processing. “Random copy-paste” denotes extracting abnormal manhole cover samples and random pasting onto the training set. “VGCopy-paste” denotes extracting abnormal manhole covers and using VGCopy-paste. AP50 and AP75 evaluation metrics are adopted from mAP.

	Method	Params	$AP_{50}\;$	$AP_{75}\;$
FastestDet	Without copy–paste	4.74 M	63.2	30.7
FastestDet	Random copy–paste	4.74 M	67.4	31.2
FastestDet	VGCopy-paste	4.74 M	**76.1**	**46.0**
YOLOv5s	Without copy–paste	7.02 M	61.9	30.9
YOLOv5s	Random copy–paste	7.02 M	78.3	41.7
YOLOv5s	VGCopy-paste	7.02 M	**80.0**	**54.6**
CenterNet-DLA34	Without copy–paste	20.17 M	64.1	22.0
CenterNet-DLA34	Random copy–paste	20.17 M	70.5	38.7
CenterNet-DLA34	VGCopy-paste	20.17 M	**70.9**	**39.9**
Retinanet-D34	Without copy–paste	31.52 M	50.5	17.8
Retinanet-D34	Random copy–paste	31.52 M	70.3	24.3
Retinanet-D34	VGCopy-paste	31.52 M	**70.7**	**25.3**
YOLOv7	Without copy–paste	37.21 M	73.4	35.9
YOLOv7	Random copy–paste	37.21 M	80.5	50.6
YOLOv7	VGCopy-paste	37.21 M	**81.8**	**56.5**

**Table 4:** Road segmentation models evaluated on the UAS test set.

	Params	meanIOU
FCN	35.31 M	91.9
UNet	4.32 M	96.5
Ours	1.17 M	**97.9**

**Table 5:** Performance of different data augmentation methods in the abnormal road manhole cover detection task.

Method	$\;\;\;\;AP_{50}\;$	$AP_{75}\;$
Mixup	60.1	33.1
Cutout	46.6	13.4
Random affine	63.1	27.8
HVS augmentation	40.8	13.1
Random copy–paste	78.3	41.7
VGCopy-paste	**80.0**	**54.6**

**Table 6:** Sensitive analysis on different hyperparameter configurations. AP50 and AP75 evaluation metrics were adopted from mAP.

No. of Pasted Samples	Model	Pasted Range	$AP_{50}\;$	$AP_{75}\;$
1	YOLOv5	0.58–0.91	79.5	49.9
1	YOLOv5	0.67–0.91	**80.0**	**54.6**
1	YOLOv5	0.77–0.91	75.4	48.9
1	YOLOv5	0.67–0.91	**80.0**	**54.6**
2	YOLOv5	0.67–0.91	79.7	51.5
3	YOLOv5	0.67–0.91	79.0	49.8
1	YOLOv7	0.58–0.91	81.6	55.8
1	YOLOv7	0.67–0.91	**81.8**	**56.5**
1	YOLOv7	0.77–0.91	80.0	54.9
1	YOLOv7	0.67–0.91	81.8	**56.5**
2	YOLOv7	0.67–0.91	**82.8**	52.5
3	YOLOv7	0.67–0.91	81.0	51.2

## Data Availability

Part of the code used in this study is freely available at an open-source version control website: https://github.com/XavierYu404/Data-Augmented-Deep-Learning-Model-for-Abnormal-Road-Manhole-Cover-Detection.git (accessed on 26 February 2023).
